# Bilateral Avascular Necrosis of the Femoral Heads in Ankylosing Spondylitis Requiring Staged Total Hip Arthroplasty: A Case Report of Diagnostic and Therapeutic Challenges

**DOI:** 10.1002/ccr3.72801

**Published:** 2026-05-26

**Authors:** Syeda Simrah Shah, Muhammad Ahsan, Tehreem Farooq, Mirza Daniyal Baig, Mirza Mohammad Ali Baig, Mohammed Hammad Jaber Amin

**Affiliations:** ^1^ Dow Medical College Karachi Pakistan; ^2^ Jinnah Sindh Medical University Karachi Pakistan; ^3^ Shaheed Mohtarma Benazir Bhutto Medical College Karachi Pakistan; ^4^ Islamic International Medical College Riphah International University Rawalpindi Pakistan; ^5^ Faculty of Medicine Alzaiem Alazhari University Khartoum Sudan

**Keywords:** ankylosing spondylitis, avascular necrosis, HLA‐B27, total hip arthroplasty

## Abstract

Ankylosing spondylitis (AS) is a chronic immune‐mediated inflammatory arthropathy primarily affecting the axial skeleton but may involve peripheral joints, particularly the hips. Avascular necrosis (AVN) of the femoral head represents a severe, underrecognized complication whose pathogenesis in AS is multifactorial—encompassing disease‐intrinsic inflammatory vasculopathy, iatrogenic glucocorticoid exposure, and, potentially, a prothrombotic state. We report the case of a 28‐year‐old HLA‐B27–positive male with a decade‐long history of AS who presented with bilateral AVN of the femoral heads superimposed on secondary hip osteoarthritis, compounded by severe microcytic anemia (hemoglobin 6.5 g/dL) and a recent COVID‐19 infection. The patient was initiated on infliximab (TNF inhibitor) for disease control and underwent staged bilateral total hip arthroplasty (left hip: March 2023; right hip: October 2024), achieving marked functional recovery. This case underscores the importance of prospective hip surveillance in AS, delineates the mechanistic nexus between systemic inflammation, corticosteroid use, and AVN, and highlights the perioperative considerations unique to biologic‐treated AS patients. A multidisciplinary rheumatology‐orthopedic approach is essential to optimize outcomes in this high‐complexity population.

## Key Clinical Message

Ankylosing spondylitis can cause severe extra‐spinal complications, including bilateral femoral head AVN, impairing function. Early recognition of hip involvement, MRI for detection, prompt biologic therapy, and timely total hip arthroplasty are crucial. A multidisciplinary rheumatology‐orthopedic approach optimizes outcomes, preserves mobility, and prevents long‐term disability in young patients with advanced disease.

## 1 Introduction

Ankylosing spondylitis (AS) is a chronic, progressive, systemic inflammatory arthritis with a strong genetic predisposition, primarily affecting the sacroiliac joints and the spine [1]. The disease is characterized by chronic inflammatory back pain, typically accompanied by morning stiffness that improves with exercise. It often manifests with musculoskeletal lesions, such as synovitis, enthesitis, and dactylitis, and extra‐musculoskeletal complications, including uveitis, psoriasis, inflammatory bowel disease, and cardiovascular involvement [2].

The pathogenesis of AS remains unclear, but a strong correlation with human leukocyte antigen (HLA)‐B27 positivity has been well established, with 80%–95% of AS patients worldwide testing positive for this marker [3]. Over time, these inflammatory changes can lead to osteitis, sclerosis, vertebral “squaring,” and the formation of syndesmophytes, culminating in the characteristic “bamboo spine” in advanced stages [4].

While spinal involvement is the most recognized feature of AS, peripheral joint involvement, particularly of the hips, contributes significantly to the disease burden. Hip involvement is seen in 25%–50% of AS patients, with up to 90% of those cases presenting as bilateral disease. This often progresses to secondary osteoarthritis (OA) in the hips, severely impairing quality of life and functional capacity [5, 6, 7]. In such cases, joint replacement surgery becomes indispensable to restore mobility and relieve pain [8].

Avascular necrosis (AVN) of the femoral head, defined as ischemic death of bone and marrow due to disrupted microvascular perfusion, represents a distinct and severe complication in AS [9]. Its occurrence in this context is incompletely understood but is believed to reflect a convergence of disease‐intrinsic mechanisms (chronic inflammation, cytokine‐driven endothelial dysfunction, and hypercoagulability), iatrogenic factors (corticosteroid use), and, potentially, intercurrent prothrombotic conditions such as SARS‐CoV‐2 infection. The simultaneous presence of AS‐associated hip arthropathy and superimposed AVN poses formidable diagnostic and therapeutic challenges, particularly in young patients where joint preservation carries paramount importance.

This report describes the case of a 28‐year‐old male with long‐standing AS who developed bilateral femoral head AVN requiring staged THA. We present a detailed analysis of the multifactorial pathogenesis of AVN in the context of AS, evaluate the diagnostic approach, and discuss perioperative management of biologic therapy. The case is intended to heighten clinical awareness of AVN as a complication of AS, advocate for systematic hip surveillance, and provide a framework for multidisciplinary management.

## 2 Case History

A 28‐year‐old married male, father of one child, presented to our rheumatology service with a 10‐year history of inflammatory back pain and a 6‐month history of progressive left hip pain. The back pain had been insidious in onset, initially confined to the dorsolumbar region, and had progressively involved the sacral area over time. It was characteristically worse with prolonged inactivity and partially relieved by movement, hot packs, and over‐the‐counter (OTC) nonsteroidal anti‐inflammatory drugs (NSAIDs). During the preceding 6–8 months, the pain had intensified in severity, acquiring a sharp, lancinating quality in the lower back and left hip, with radiation down the left lower limb accompanied by paresthesia. Morning stiffness exceeding 1 h was reported.

Constitutional symptoms included low‐grade fever, significant unintentional weight loss, and generalized myalgia. Gastrointestinal symptoms comprised recurrent central abdominal pain, constipation, and intermittent hematochezia, attributed to chronic NSAID use. The patient also reported xerophthalmia (dry eyes), recurrent oral aphthous ulcers, and dysuria. There was no history of uveitis, genital ulcers, psoriatic skin lesions, chest pain, or dyspnea. Notably, the patient reported a prior episode of COVID‐19 infection (exact timing not documented). He had been prescribed a short course (approximately 1 week) of OTC glucocorticosteroids of unspecified type and dose for acute pain management; the precise dates were not retrievable at the time of evaluation. He had received four units of packed cell volume (PCV) transfusion 1 year prior for anemia of undetermined etiology. Family history was unremarkable for rheumatologic or hematologic disease. He was a light smoker and habitually chewed betel nut; dietary history suggested low intake of red meat and dairy products.

Physical examination revealed a thin, alert, and oriented male. Hemodynamic parameters demonstrated hypotension (blood pressure 90/60 mmHg) and tachycardia (pulse 96 bpm); he was afebrile. Musculoskeletal examination revealed bilateral sacroiliac joint tenderness, loss of lumbar lordosis, and globally restricted lumbosacral movements. Left hip examination demonstrated severe tenderness with markedly limited range of motion in all planes. Oral examination confirmed multiple aphthous ulcers. Cardiovascular, respiratory, abdominal, and neurological examinations were unremarkable apart from the aforementioned findings.

## 3 Differential Diagnosis, Investigations and Treatment

### 3.1 Differential Diagnoses

The working differential diagnosis encompassed ankylosing spondylitis (primary consideration), enteropathic arthritis (given GI symptoms), spinal tuberculosis (endemic region; constitutional features), Behçet's disease (oral ulcers, GI involvement), reactive arthritis, and osteomalacia (dietary deficiency, poor sunlight exposure). Rheumatoid arthritis and psoriatic arthritis were considered but deemed unlikely given the seronegative profile and clinical picture.

### 3.2 Investigations

#### 3.2.1 Laboratory Findings

HLA‐B27 was positive. Autoimmune markers (ANA, rheumatoid factor, anti‐CCP antibodies) were negative. Complete blood count demonstrated microcytic hypochromic anemia: hemoglobin 6.5 g/dL, MCV 61.3 fL, and RDW 18.5%. Inflammatory markers were elevated (ESR 80 mm/h; CRP 75 mg/L), consistent with active inflammatory disease. Renal and hepatic function tests were within normal limits.

Pre‐biologic screening was performed prior to infliximab initiation: interferon‐gamma release assay (IGRA) for latent tuberculosis, hepatitis B surface antigen (HBsAg), and anti‐hepatitis C virus antibody (anti‐HCV) were all negative. Antiphospholipid antibodies (aPL), a thrombophilia panel (protein C, protein S, antithrombin III, Factor V Leiden, prothrombin gene mutation), and a fasting lipid profile were not obtained due to financial constraints.

#### 3.2.2 Imaging

**FIGURE 1:**
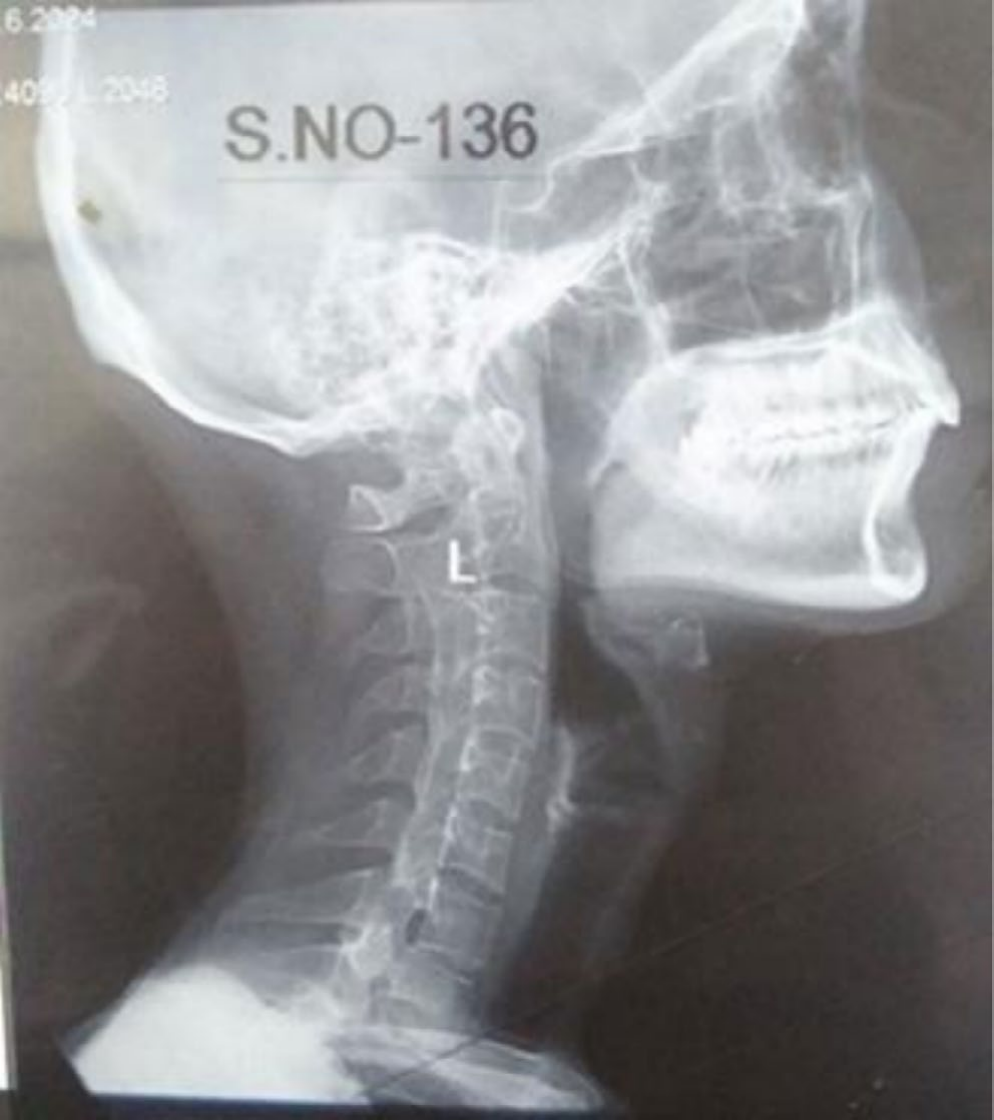
Cervical spine showing vertebral squaring and loss of lordosis.

**FIGURE 2:**
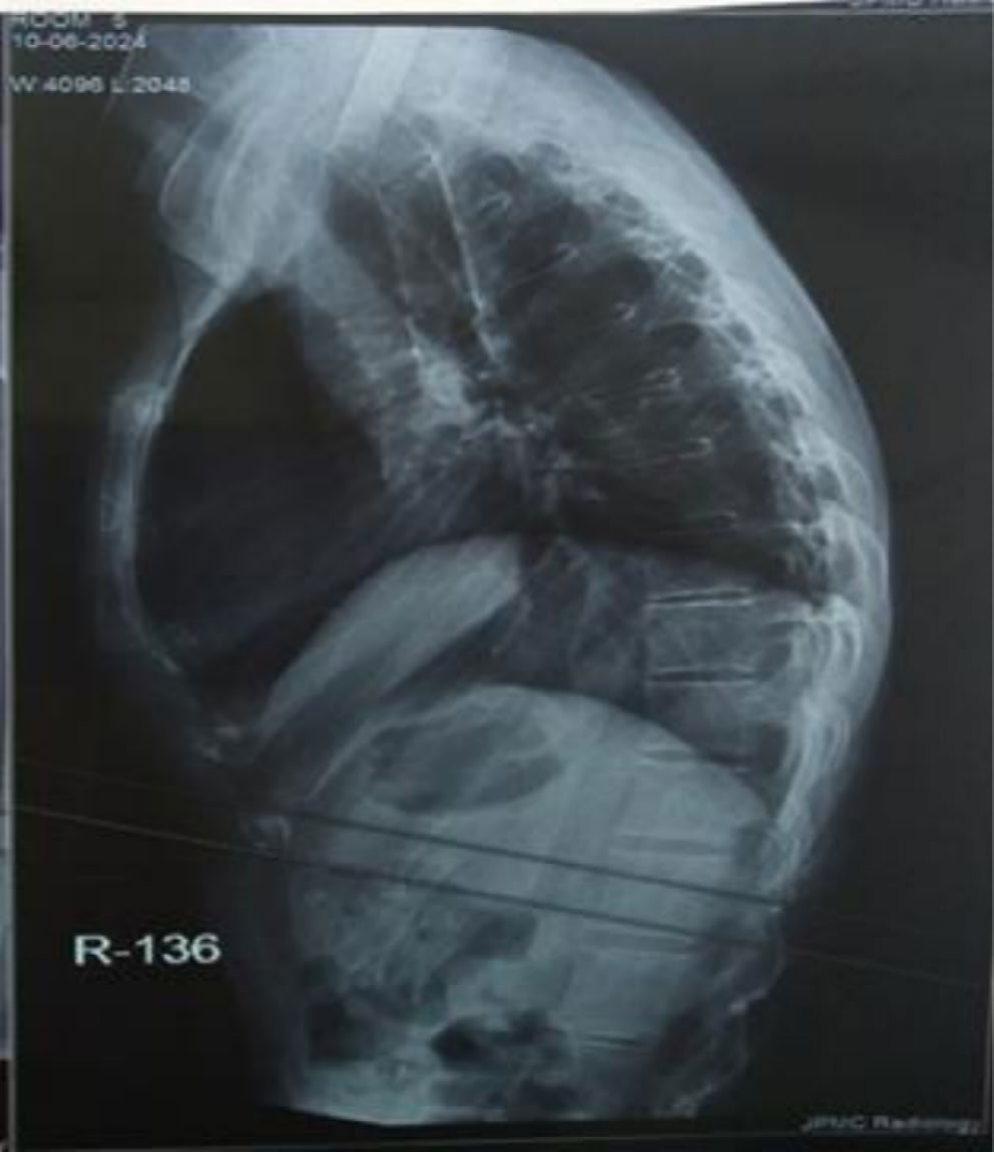
Dorsal and lumbar spine shows squaring of vertebrae with reduced disc spaces. Also, mild sclerotic margins are seen.

**FIGURE 3:**
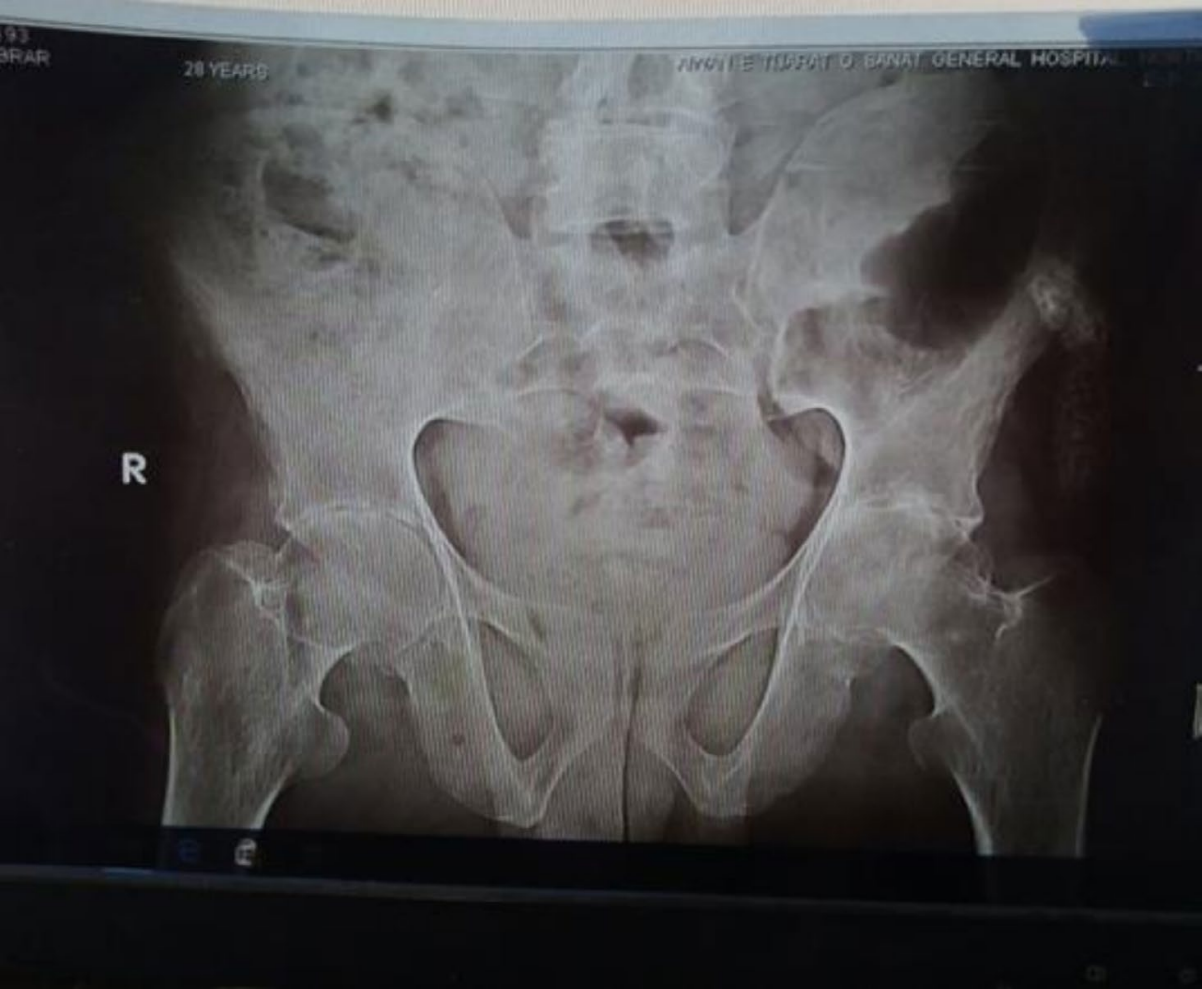
Partial fusion of bilateral sacroiliac joints, more on the right side along with bilaterally reduced hip joint space with sclerosis. Sclerosis noted along the superio‐medial aspect of the left femoral head, suggestive of AVN of the left femoral head.

Radiological investigations are as follows:

The cervical spine X‐ray demonstrated vertebral squaring and loss of lordosis (as shown in Figure 1). The dorsal and lumbar spine X‐rays revealed squaring of the vertebrae, reduced intervertebral disc spaces, and mild sclerotic margins, which are consistent with chronic inflammatory changes typically observed in AS (as shown in Figure 2). The pelvic X‐ray showed partial fusion of sacroiliac joints bilaterally, more pronounced on the right, along with reduced hip joint spaces and sclerosis on both sides. Additionally, sclerosis along the superomedial aspect of the left femoral head was observed, suggestive of AVN of the left femoral head (as shown in Figure 3).

MRI of the spine confirmed bilateral sacroiliitis and fibrotic changes at affected joints. It also shows signs of active sacroiliitis, including bone marrow edema and subchondral sclerosis adjacent to S1 joints. Bilaterally reduced hip joint space is seen along with sclerosis in the superomedial aspect of the left femoral head, consistent with AVN. MRI of the hips revealed subchondral signal abnormalities bilaterally, particularly on the left side, indicative of Stage C AVN according to Mitchell's classification. Mitchell's MRI classification for AVN defines stage C as involving more than 30% of the femoral head with subchondral fractures (crescent sign) and early femoral head deformity. This staging highlights the advanced disease state, necessitating timely intervention to prevent further joint damage. Trace bilateral joint effusions were also noted. No signs of marrow replacement or significant paraspinal soft tissue involvement were evident.

### 3.3 Treatment

#### 3.3.1 Medical Management

The patient was initiated on infliximab (biosimilar), a tumor necrosis factor (TNF) inhibitor, with an induction dose of 225 mg administered at 0, 2, and 4 weeks, followed by maintenance therapy every 4 weeks. This biologic therapy was selected based on the patient's high Bath Ankylosing Spondylitis Disease Activity Index (BASDAI) score of 7.9, indicating severe disease activity. In addition to the primary treatment, the patient was provided with vitamin D3 and calcium supplements to support bone health, pregabalin to ease nerve pain, celecoxib to manage his discomfort, and folic acid to improve his nutritional status. Moreover, two units of blood were transfused to address his anemia. Although the patient had anemia and mild GI symptoms, Esophagogastroduodenoscopy (EGD) was not performed given: (1) absence of overt active hemorrhage; (2) hemodynamic stabilization post‐transfusion; (3) clinical prioritization of AS management and surgical planning; (4) resource constraints. Prospective endoscopic evaluation is recommended at follow‐up. Infliximab was withheld in the perioperative period for both stagged THA procedures and resumed following wound healing confirmation.

#### 3.3.2 Surgery

Due to the severity of hip involvement and the diagnosis of bilateral avascular necrosis (AVN) of the femoral heads, the patient was referred to the orthopedic department. Under their care, the patient underwent total hip replacements (THR), first on the left hip in March 2023, followed by the right hip in October 2024. Both surgeries were performed successfully, significantly improving the patient's mobility and alleviating hip pain. The procedures were uneventful, and the patient reported marked improvement in his ability to perform daily activities postoperatively.

## 4 Conclusion and Result (Outcome and Follow‐Up)

The combination of serological and imaging findings confirmed the final diagnosis of ankylosing spondylitis with bilateral avascular necrosis of the hip joints, facilitating a targeted treatment approach.

Post‐surgery, the patient was enrolled in a structured rehabilitation program. He is following a regular physiotherapy schedule to strengthen the surrounding muscles, improve joint range of motion, and accelerate recovery. Weight‐bearing exercises were introduced gradually, and compliance with physiotherapy has contributed to significant functional improvements.

The patient remains under close follow‐up with both rheumatology and orthopedic teams. He continues on maintenance infliximab therapy, which has helped control systemic inflammation, as evidenced by the marked decline in ESR and CRP levels. On follow‐up, he reported significant relief from back and hip pain, enhanced general well‐being, improved appetite, and increased mobility. Periodic reviews by the orthopedic team are ongoing to monitor his recovery and ensure the longevity of the prosthetic joints.

## 5 Discussion

AS is diagnosed based on a combination of clinical, laboratory, and imaging findings that align with axial spondyloarthritis [10]. He modified New York criteria require at least one clinical feature—inflammatory back pain, limited lumbar mobility, or restricted chest expansion—paired with radiographic sacroiliitis. These criteria have low sensitivity for early disease; sacroiliitis may not be visible on standard radiographs for up to 9 years, and MRI of the sacroiliac joints should be used when early disease is clinically suspected [11, 12].

The differential diagnosis includes lumbar disc disease, osteoarthritis, rheumatoid arthritis, psoriatic arthritis, reactive arthritis, and diffuse idiopathic skeletal hyperostosis [10, 11, 12, 13]. In this patient, HLA‐B27 positivity, axial inflammatory pain, MRI‐confirmed sacroiliitis, and vertebral squaring established AS; the seronegative autoimmune profile and imaging distinguished it from these alternatives.

Bilateral hip involvement secondary to AS was further complicated by AVN of the femoral heads, creating a significant diagnostic and therapeutic challenge. THA provided substantial pain relief and functional improvement consistent with published evidence, though long‐term success depends on ongoing disease control, spinal involvement, and extra‐articular disease activity [14, 15].

The pathogenesis of AVN in AS is multifactorial. Chronic synovial inflammation—driven by TNF‐α, IL‐6, and IL‐17—promotes endothelial injury, coagulation dysregulation, and intraarticular hypertension, all of which compromise femoral head perfusion [9]. Corticosteroid use, even short‐course, accelerates marrow adipogenesis and raises intraosseous pressure, impairing the sinusoidal microcirculation. In this patient, an approximately one‐week OTC glucocorticoid course cannot be excluded as a contributing factor. SARS‐CoV‐2 infection is an emerging independent risk factor for AVN, mediated by ACE2‐driven endothelial injury and a procoagulant state [16]. The patient's prior COVID‐19 infection is a biologically plausible additional mechanism, though its temporal relationship to AVN onset could not be established.

The published literature on AVN complicating AS is sparse. Stoicănescu et al. (2019) reported bilateral femoral head AVN in an AS patient with concurrent meningioma and Hodgkin's lymphoma—independent prothrombotic conditions that complicate direct aetiological comparison [17]. Our patient presented without malignancy but with COVID‐19 history and OTC glucocorticoid exposure as distinct risk factors. Both cases share bilateral femoral head involvement and the rare coexistence of AS and AVN; the younger presentation (28 years) and successful staged THA with biologic management distinguish our case and extend the documented spectrum of this association.

aPL antibodies, a thrombophilia panel, and a fasting lipid profile were not obtained due to financial constraints. This is a significant limitation: antiphospholipid syndrome, inherited thrombophilias, and dyslipidemia each independently cause AVN and may have amplified the mechanisms above. These investigations are strongly recommended in future similar cases, particularly in young patients.

Infliximab was selected on the basis of BASDAI 7.9 and NSAID failure. Pre‐biologic screening—IGRA, HBsAg, and anti‐HCV—was negative before initiation. Infliximab was withheld at least 4 weeks before each THA and resumed four to 6 weeks postoperatively following confirmed wound healing, consistent with ACR/AAHKS 2017 perioperative guidance.

This case underscores the importance of MRI‐based hip surveillance in AS patients with long disease duration, high inflammatory burden, or corticosteroid exposure. A coordinated rheumatology‐orthopedic approach, with careful perioperative biologic management, is essential to achieve functional recovery in this population.

## 6 Limitations

This case is limited by its single‐patient design and lack of generalizability. The etiology of anemia and contributing factors to avascular necrosis were not fully evaluated. Early imaging was unavailable, limiting assessment of disease progression. Second, the steroid preparation, dose, and exact duration of use could not be retrieved, limiting precise quantification of this iatrogenic risk factor. Third, the temporal relationship between COVID‐19 infection and AVN onset cannot be established from available documentation, precluding formal causal attribution; the COVID‐19 contribution is therefore presented as a biologically plausible hypothesis rather than a confirmed etiological factor. Fourth, long‐term prosthetic outcomes beyond the current follow‐up period are unknown, and extended surveillance will be required to assess implant longevity and disease trajectory. These limitations do not invalidate the diagnostic conclusions or the principal clinical messages of the report; they are transparently disclosed to contextualize the findings and identify directions for future investigation.

## 7 Conclusion

This case underlines the importance of early hip MRI surveillance, prompt biologic therapy for disease control, and coordinated rheumatologic‐orthopedic management. Staged bilateral THA with appropriate perioperative biologic management can significantly improve function and quality of life, especially in young patients.

## Author Contributions

**Mirza Daniyal Baig:** writing – original draft, writing – review and editing, methodology, project administration, supervision, data curation. **Muhammad Ahsan:** writing – review and editing, writing – original draft, methodology, resources. **Syeda Simrah Shah:** conceptualization, writing – original draft, writing – review and editing, validation, project administration, supervision. **Tehreem Farooq:** writing – original draft, visualization, investigation, software. **Mohammed Hammad Jaber Amin:** writing – review and editing. **Mirza Mohammad Ali Baig:** writing – review and editing, writing – original draft, validation.

## Funding

The authors have nothing to report.

## Disclosure

Permission to Reproduce Material From Other Sources: All figures, tables, or text excerpts reproduced from previously published material are properly cited, and permission has been obtained from the copyright holders where required.

## Ethics Statement

This case report was conducted in accordance with the ethical standards of the institutional review board. Formal approval was waived for this retrospective case study as per institutional guidelines.

## Consent

Written informed consent was obtained from the patient and legal guardian for the publication of this case report, including de‐identified clinical details and images.

## Conflicts of Interest

The authors declare no conflicts of interest.

## Data Availability

The data that support the findings of this study are available on request from the corresponding author. The data are not publicly available due to privacy or ethical restrictions.
